# Papillary Thyroid Carcinoma: An Autobiographical Case Report

**DOI:** 10.7759/cureus.22559

**Published:** 2022-02-24

**Authors:** Joshua C Hunsaker, Greg Hoffman

**Affiliations:** 1 Medical School, University of Utah Hospital, Salt Lake City, USA; 2 Family Medicine, Intermountain Healthcare, Sandy, USA

**Keywords:** levothyroxine, radioactive iodine, thyroid malignacy, papillary carcinoma of thyroid, thyroid cancer

## Abstract

Papillary thyroid carcinoma (PTC) is the most common malignant thyroid neoplasm with the median age at presentation for papillary carcinoma being around 50 years. This case report describes the author’s experience of being diagnosed with PTC at the age of 25, as well as the course of treatment, and eventual outcome.

## Introduction

Carcinoma of the thyroid gland accounts for approximately 1% of all newly diagnosed malignant diseases and is predominantly found in females (3:1) [1,2]. These carcinomas are commonly classified as papillary, follicular, medullary, or anaplastic carcinomas. Papillary carcinomas are considered “well-differentiated” and are responsible for between 80-85% of all thyroid malignancies [1-3]. The median age at presentation for papillary carcinoma is 50 years.

The most common presentation of papillary carcinoma of the thyroid is an asymptomatic (painless) mass at the level of the thyroid. In around 20% of cases, patients may present with dysphagia or hoarseness which likely indicates involvement of the recurrent laryngeal nerve and/or tracheal compression [1,4]. These patients will often have normal thyroid function testing. The diagnosis is made with ultrasound and fine-needle aspiration (FNA) [4]. Definitive treatment is surgical intervention with total thyroidectomy or lobectomy if the tumor is noted to be unifocal and < 4 cm without evidence of lymph node metastasis. In those with advanced primary tumors, unilateral or bilateral neck dissection is indicated based on severity to evaluate the extent of local lymph nodes and for further staging [1,2,4]. Furthermore, neck dissections can be lateral compartment dissections or central compartment dissections, both of which have their own set of indications that are beyond the scope of this case report.

This case report describes the author’s experience of being diagnosed with PTC at 25 years of age, including a description of symptom onset along with subsequent workup and treatment. Throughout this case report, “I” will refer to myself, Joshua Hunsaker. I believe there is great value in narrative medicine and my hope is that those who are diagnosed with PTC will have a better understanding of what to expect throughout their workup and treatment.

## Case presentation

**Figure 1:**
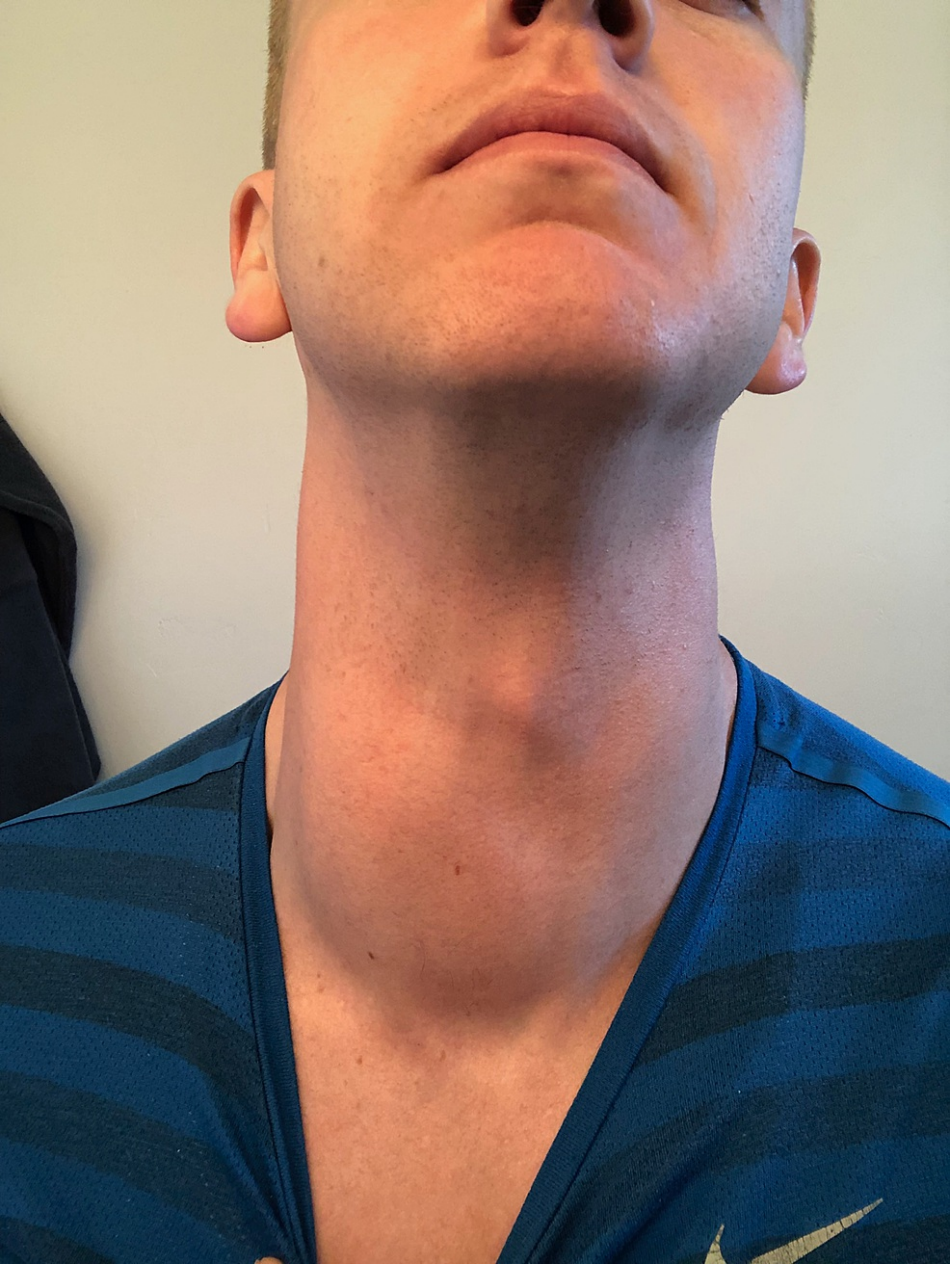
Initial Presentation of Right-Sided Neck Mass

At 25 years of age, I was less than two weeks from graduating from my undergraduate institution and had already accepted an offer to attend medical school at the University of Utah in three short months. Other than occasional episodes of exercise-induced asthma, I was healthy and extremely active. In preparation for a wedding, I found myself putting on a collared shirt that had fit very well just a few days prior. Upon securing the top button, I found myself struggling to breathe and released the button to take a closer look at my neck. On inspection, I noted a large visible outline on the right side of my neck near the level of the thyroid that I had not visualized previously (Figure 1). Two days later, I was seen by a local ENT who was conservative in his initial workup due to the acute onset of the mass, which made malignancy less likely. I was treated with a five-day course of prednisone with instructions to return if the mass did not decrease in size. After no change was noted following the five-day course of steroids, thyroid function tests were ordered in the form of TSH and free T4 as indicated to rule out autoimmune or infectious etiologies that are common in thyroiditis [5]. Thyroid ultrasound and fine-needle aspiration were also ordered for possible malignancy workup [1-3].

Findings

Results of TSH and free T4 blood tests were both within normal limits, which is common in thyroid malignancies [1]. Ultrasound findings included an 8.1 x 4.9 x 6.3 cm solid mass occupying essentially the entire right thyroid lobe for which FNA was indicated. Fine needle aspiration of the mass was histologically consistent with papillary thyroid carcinoma and surgical intervention was indicated. Less than a week after not being able to button my shirt, I was told I had cancer and was scheduled for surgery.

Surgery

**Figure 2:**
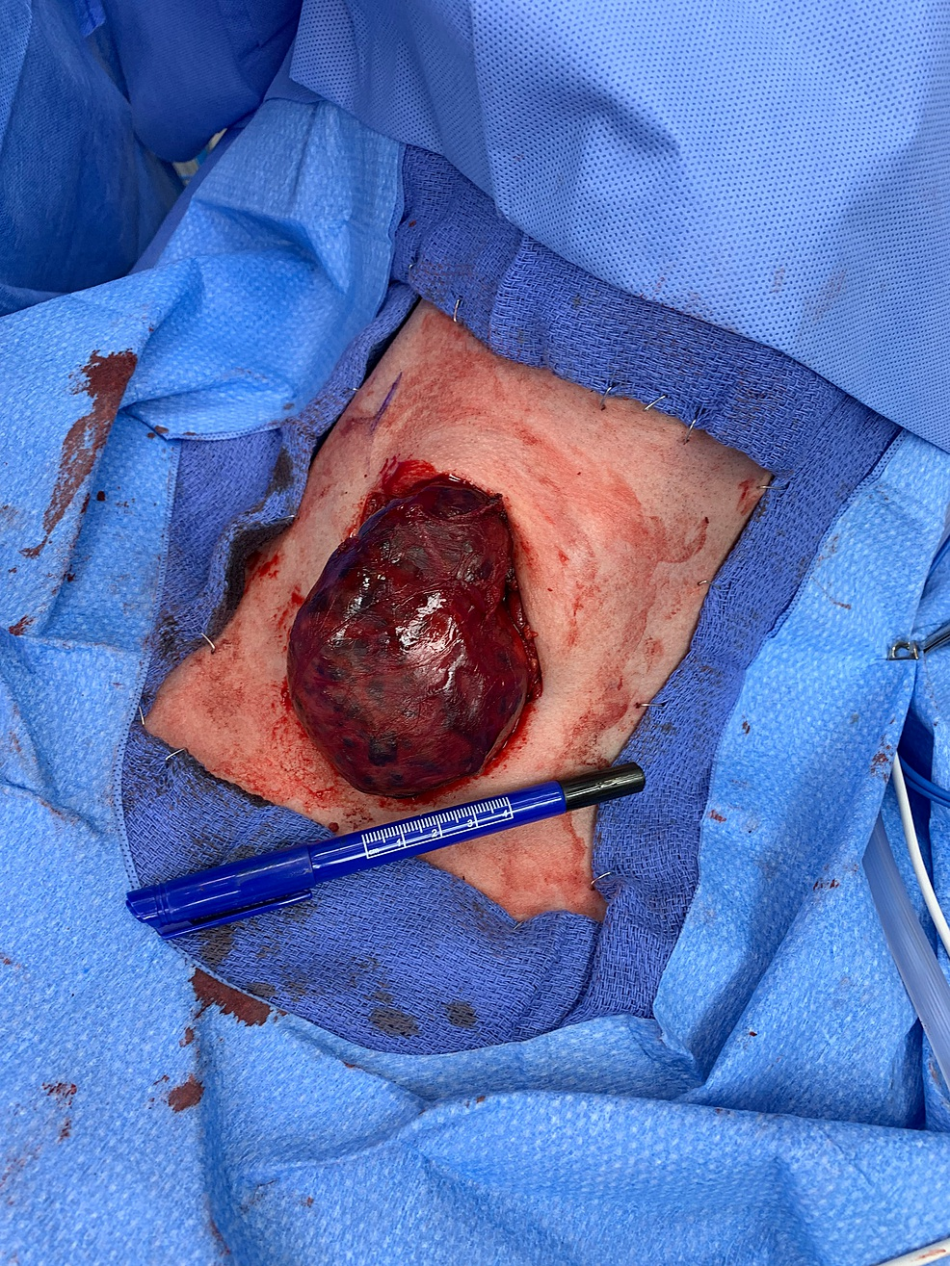
Intraoperative View of the Malignant Right Thyroid Nodule

**Figure 3:**
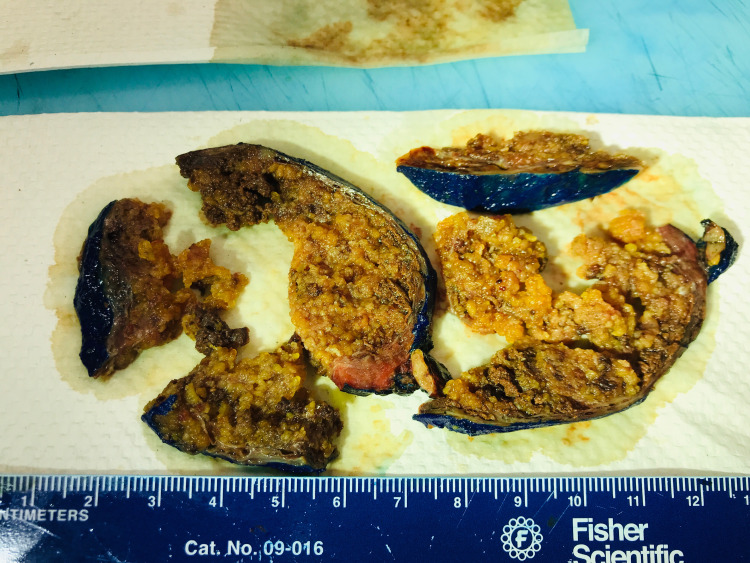
Gross Pathology Sections of the Resected Thyroid Papillary Carcinoma

Total thyroidectomy with elective right neck dissection was planned due to the advanced size of the primary tumor and suspected lymph node involvement which is extremely common in PTC [1,2,4]. The primary tumor was first identified intraoperatively as seen in (Figure 2) and was sent to pathology for frozen as well as permanent sections as seen in (Figure 3). All margins of the primary tumor were noted to be negative. Following right neck dissection, lymph nodes were sent to pathology and 31/56 were positive for metastatic papillary carcinoma but there was no immediate sign of extranodal extension. Following current guidelines, tumor staging was indicated. The primary tumor was staged as pT3a. pT3 tumors of the thyroid are defined as differentiated thyroid cancers measuring more than 4 cm in their greatest dimension that are limited to the thyroid with minimal extrathyroidal extension [6]. The final staging was designated as pT3a, pN1b, MX. Due to the extent of lymph node involvement, radioactive iodine treatment was indicated. Despite the young age of the patient and the rather advanced disease, molecular testing was deferred.

Radiation treatment and long-term follow up

One week after being discharged from the hospital, I met with radiation oncology and was informed of the treatment options for my specific cancer as well as the current plan. The standard of care for radiation treatment following thyroidectomy in patients with advanced primary tumors or high-risk staging is with radioactive iodine-131 ablation [6-8]. I was given instructions to avoid foods and additives that contained iodine for two weeks in order to starve my body of iodine, which would make the treatment more effective. Following treatment with I-131, I was instructed to isolate myself in my room for one week in order to avoid transmitting potential radiation effects to my roommates. During this time, I also suffered from a transient ageusia that lasted for three weeks. Transient ageusia is reported by almost 30% of patients following I-131 treatment [9]. I was started on a low dose of levothyroxine and had thyroid function labs drawn every two weeks until my TSH was sufficiently suppressed with a T4 level that was within normal limits. To monitor for recurrence, I initially had thyroglobulin (TBG) levels checked every three to six months for the first two years postoperatively and now have the level checked every year as the primary literature indicates [10].

## Discussion

This case report describes a unique presentation of papillary carcinoma of the thyroid due to the less than ordinary demographics of the patient. PTC is much more common in women (between 2:1 and 4:1) and has a median age at presentation of 50 years [1,2]. The patient described was an otherwise healthy male who presented at the age of 25 years old. Also, in PTC, the tumor size is often between 1-3 cm but in this case, the patient had a tumor that measured over 8 cm [11,12].

Girardi et al. performed a retrospective analysis to analyze the clinicopathological pattern of thyroid carcinoma presentation according to age groups. Their study included 596 patients who underwent thyroidectomy with curative intent. The patients were grouped according to age and were compared to identify potential differences in gender as well as histological, and pathological characteristics. They found that younger individuals had the highest prevalence of neurovascular invasion, capsular invasion, and lymph node metastasis. They were also noted to have a larger tumor diameter and higher rate of extra glandular disease [13]. These characteristics fit with the clinical picture described in my case report. Despite having more aggressive and advanced tumors, however, younger patients also had higher survival rates than the older groups [13]. This indicates that advanced age is likely an important risk factor for the overall survival rate in thyroid carcinoma.

Well-differentiated thyroid malignancies are often described as “the ones you want,” due to their low rates of distant metastasis and highly specific treatments along with high five-year survival rates. Following surgical resection of well-differentiated thyroid carcinomas, those with significant lymph node involvement or primary tumor size >4 cm should undergo radiation therapy with the radioactive isotope Iodine-131. This form of radiation is highly selective for any remaining thyroid cells as they are the only cell type in the body that use iodine. There are, however, differing opinions related to the amount of I-131 that should be given to patients in order to decrease the risk of side effects of radiation. Shengguang et al. performed a systematic review of the literature to compare the success rates of I-131 for remnant ablation following thyroidectomy. They found that higher doses of I-131 (100mCi), resulted in higher rates of successful ablation when compared to lower doses (30mCi) in the treatment of low-risk differentiated thyroid carcinomas [8]. In response to the fear of added side effects with higher doses of I-131, Kaewput et al. performed a retrospective review of 176 patients who received a cumulative dose of >600mCi following thyroidectomy and found complications attributed to the radiation in only 14% of cases, of which only 3.9% were permanent salivary gland dysfunction [14].

Levothyroxine is the standard for thyroid hormone replacement therapy following thyroidectomy. The target range for TSH levels in those on thyroid replacement therapy has been a topic of discussion for years. Pujol et al. retrospectively analyzed 141 patients who were being managed with thyroid replacement therapy following thyroidectomy and found that a greater degree of TSH suppression was an independent predictor of prolonged length of relapse-free survival [15]. This indicates that if a patient is able to tolerate supratherapeutic levels of thyroid replacement, they are more likely to remain cancer-free.

## Conclusions

This autobiographical case report illustrates the typical workup for undiagnosed thyroid masses as well as the typical treatment course for thyroid carcinomas, including the use of I-131 following surgical resection. While PTC can present in a number of ways and across a diverse patient population, the general treatment course tends to be similar in the majority of cases. This algorithmic approach based on peer-reviewed literature helps to take the guesswork out of the proper treatment course for not only providers but patients as well.
